# IL-23 Inhibition in Ankylosing Spondylitis: Where Did It Go Wrong?

**DOI:** 10.3389/fimmu.2020.623874

**Published:** 2021-02-18

**Authors:** Dominique Baeten, Iannis E. Adamopoulos

**Affiliations:** ^1^ Clinical Immunology and Rheumatology, Amsterdam University Medical Center, Amsterdam, Netherlands; ^2^ Immunology Therapeutic Area, UCB, Slough, United Kingdom; ^3^ Department of Medicine, Division of Rheumatology and Clinical Immunology, Beth Israel Medical Deaconess Center, Boston, MA, United States

**Keywords:** interleukin-23, interleukin-17, ankylosing spondylitis, axial spondyloarthritis, Th17

## Abstract

Axial spondyloarthritis is a prevalent form of chronic arthritis which is related to psoriatic arthritis and skin psoriasis. TNF and IL-17A as well as IL-17F are key cytokines contributing to the pathobiology of this disease, as evidence by the therapeutic efficacy of inhibition of these factors. Despite the evidence that IL-23 acts as an upstream driver of Th17 cells, the T lymphocytes producing IL-17, and that IL-23 inhibition shows profound efficacy in psoriasis, blocking IL-23 failed to show any evidence of clinical efficacy in axial spondyloarthritis. In this viewpoint article, we revisit the reasons-to-believe in a role of IL-23 in the pathobiology of axial spondyloarthritis, discuss what we have learned on the pathobiology of this disease in general and on the function of the IL-23/IL-17 axis in particular, and share a handful of lessons learned that are of relevance for the translation of emerging biological insights into clinical therapeutics.

## Introduction

Axial spondyloarthritis (AxSpA) is a prevalent form of chronic arthritis affecting mainly the axial skeleton (1). As other forms of spondyloarthritis, including psoriatic arthritis, it also often affects peripheral joints. Both axial and peripheral disease is characterized by a combination of chronic inflammation (including synovitis, enthesitis, and osteitis), focal bone destruction, and exaggerated pathological new bone formation leading to joint ankylosis. Finally, a significant proportion of patients also display extra-articular manifestations such as psoriasis, Crohn’s disease or colitis ulcerosa, and acute anterior uveitis.

The pathobiology of AxSpA remains incompletely understood but a few concepts have been firmly established. First, AxSpA does not display the prototypical features of classical autoimmune diseases such as female predominance, genetic association with MHC class II variants and molecules involved in T- or B-lymphocyte activation, presence of autoantibodies, and good clinal response to T- or B-cell targeted therapies. It is therefore considered as a hyperinflammatory disorder driven by an abnormal inflammatory (potentially innate immune) response to different forms of stress leading to uncontrolled tissue inflammation and damage (2). Second, AxSpA is strongly associated with HLA-B27 and overexpression of human HLA-B27 in rats leads to AxSpA-like disease (3). HLA-B27 could contribute to the pathobiology of the disease by antigen-presentation to cytotoxic T cells, intracellular misfolding leading to endoplasmic reticulum stress and abnormal cytokine production (including IL-23), and/or formation of heavy chain homodimers which can directly trigger NK and T cells and possibly other cell types to produce cytokines such as IL-17 (4). Finally, pro-inflammatory cytokines such as TNF and IL-17 are critical drivers of the chronic inflammation as demonstrated by clinical efficacy of drugs blocking these cytokines. Within the IL-17 family, several IL-17A blockers have proven impact on chronic inflammation in AxSpA; preliminary evidence also suggests that IL-17A blockade may be more effective than TNF inhibition in halting pathological new bone formation (5, 6). More recently, IL-17F has been proposed to contribute beyond IL-17A in the pathobiology of both inflammation and new bone formation in spondyloarthritis (7–9). Also, other cytokines produced by so-called Th17 cells, including GM-SCF, have been implicated in the pathobiology of SpA (10), raising the question of the therapeutic value of targeting upstream activators of Th17 cells rather than IL-17 itself.

This concept has been amply explored in contrived *in vitro* and animal models, where IL-23 has been identified as a key factor in the differentiation, activation, and pathogenicity of Th17 cells (11). More importantly, drugs targeting either the IL-23–specific p40 subunit or the p19 subunit which is shared between IL-23 and IL-12 have shown impressive efficacy in skin psoriasis (12). Head-to-head studies even demonstrated the superiority of IL-23 inhibition over IL-17 inhibition in this disease, in line with a “cascade model” where “upstream” IL-23 drives downstream effector cytokines including but not restricted to IL-17A.

## IL-23 Inhibition in Axial Spondyloarthritis: What the Clinical Trials Taught Us

Considering the clinical and pathobiological link between psoriasis and spondyloarthritis and the efficacy of IL-17 blockade in both conditions, a randomized, placebo-controlled phase II clinical trial assessed the safety and efficacy of the anti-p19 antibody risankizumab in ankylosing spondylitis, the prototypical subform of AxSpA (13). Whereas no safety or intolerance signals were identified, the study failed to show any evidence for clinically significant improvement of the primary and secondary endpoints. Several lines of evidence concord to indicate that the unexpected outcome of this PoC trial is indeed true. First, the patient population is comparable to the patient populations included in other AS trials such as the anti-IL-17A trials. Second, the design of the trial including the endpoints are also well aligned with other AS trials. Third, the active drug, risankizumab, has proven efficacy in psoriasis and its PK profile was as expected. Fourth, primary, secondary, and exploratory endpoints consistently indicated lack of efficacy, and there was not even a trend toward a dose-response. Finally, a subsequent study testing the anti-p40 drug ustekinumab, which has also proven efficacy in psoriasis, also failed in ankylosing spondylitis (14). Taken together, these data provide a wealth of human pharmacological evidence that IL-23 may not be a relevant driver of the pathobiology and clinical symptoms of active, established AxSpA.

## IL-23 and Axial Spondyloarthritis: Revisiting the Evidence

This unexpected clinical finding made us revisit the scientific reason-to-believe in IL-23 blockade in AxSpA. Beyond the previously mentioned understanding of the basic biology of the IL-23/IL-17 axis and the similarities and overlap between psoriasis and AxSpA, there were three main lines of evidence that had been considered. First, genome-wide association studies (GWAS) studies have clearly established that SNPs in the IL-23R are a susceptibility factor for ankylosing spondylitis (15) and several other genetic risk factors associated with ankylosing spondylitis also point toward the IL-23/IL-17 axis. However, the relative risk of these associations is moderate to low, several variants of IL-23R are associated with different diseases (including ankylosing spondylitis, psoriasis, Crohn’s disease and ulcerative colitis), and the functional consequences of these variants remain incompletely understood. Second, over-expression of IL-23 in mice was reported to induce a spondyloarthritis-like phenotype by acting on RORgamma+ CD3+ CD4− CD8− entheseal T cells (16). However, this finding turned out to be hard to reproduce by other labs. On the contrary, we had demonstrated previously that systemic IL-23 exposure induced chronic arthritis, severe bone loss, and myelopoiesis in the bone marrow and spleen, which resulted in increased osteoclast differentiation and systemic bone loss (17), a phenotype which is not compatible with AxSpA. Third, there was indirect clinical evidence which, unfortunately, is methodologically flawed. A pilot study reported profound efficacy of the anti-p40 antibody ustekinumab in AS (18), but the uncontrolled, open-label design is completely flawed in a disease such as AS where the clinical outcomes are largely patient and physician dependent. Similarly, studies with ustekinumab in psoriatic arthritis reported a significant improvement in the BASDAI, a well-validated patient-reported outcome used in ankylosing spondylitis trials, and concluded that this drug was also effective for the axial symptoms in this disease. However, these studies ignored completely the fact that BASDAI, albeit having been developed for AS, does not in any way capture specifically axial disease and in fact is even a very good patient-reported outcome for peripheral arthritis (19).

In brief, the supporting evidence to believe in a central role for IL-23 in the pathobiology of AxSpA was, at best, circumstantial. More specifically, there is a striking lack of functional data to underpin if and how IL-23 contributes to the pathobiology of AxSpA. Studies with targeted therapies across an array of inflammatory conditions have indeed taught us that a single pathway or even cytokine with well understood basic biology can function in completely different way depending on the exact contact and thereby can drive clearly distinct pathobiology (20). In line with this, inhibition of IL-23 in collagen-induced arthritis and SKG mice ameliorated experimental arthritis but did not abolish pathology suggesting that other pathways remain active (21, 22). Besides the IL-23/IL-17 axis, we have now also demonstrated this for TNF: whereas the soluble form of TNF, signaling exclusively through the TNF-R1, drives profound synovitis and bone destruction reminiscent of what we observe in human RA, the transmembrane form of the same cytokine drives osteoproliferative axial and peripheral joint inflammation as seen in SpA (23). Similarly, mutations in specific molecules of the IL-1 pathway lead to quite distinct autoinflammatory syndromes such as DIRA and CAPS, which affect other tissues and organs (24). All these examples highlight the importance to understand not only the basic biology of the cytokine pathway, but also to decipher its exact function and relative contribution in a particular disease context.

## Revisiting the Pathobiology of AxSpA

What have we now learned on the functional role of IL-23 in AxSpA? Two recent findings deserve further exploration in this context. First, could it be that IL-23 contribute to the disease initiation but becomes redundant in established disease? In other words: could IL-23 contribute to derail an IL-17 response which, once evolved to a state of chronic inflammation, persists even in the absence of IL-23? This concept is supported by our findings in the HLA-B27 transgenic rat model of SpA. The phenotype, histology, and pathobiology of this model recapitulates faithfully human SpA, is driven by the major genetic risk factor of human AS (HLA-B27) and responds well to both TNF and IL-17 blockade (25). Targeting IL-23R in this model lacked any efficacy in a therapeutic setting but did partially prevent disease onset in a prophylactic setting (26). Albeit intriguing from a scientific angle, this hypothesis may not be helpful from a clinical angle as it is at present impossible to capture and diagnose AxSpA patients in the early or even preclinical phase.

A second major insight is that IL-17 is not only produced by canonical Th17 cells but also by a variety of innate lymphocytes including MAT cells, gamma delta T cells, iNKT and ILC3 cells (27, 28). Those cell populations have been suggested to be less dependent on IL-23 for their IL-17 production: albeit IL-23 can certainly drive IL-17 production in these cells, other cytokines such as IL-1 and IL-18 are even more potent and IL-23 appears to be redundant in the presence of these other cytokines (29). This observation fits also with the fact that those innate immune cells were recently shown to amplify myelopoiesis *via* GM-CSF (30) and M-CSF signaling (31), which diversify the pathological signals of IL-23, extend them to other pathways including IL-18, and thereby render inhibition of IL-23 less effective in established disease. There is little information on the potential role of IL-18 in AxSpA. In contrast, multiple studies have demonstrated the association of IL1 gene cluster polymorphisms with AS, in particular polymorphisms in IL-1R2 (15) and IL-1A (32). Small scale proof-of-concept clinical trials with anakinra, a soluble IL-1 decoy receptor construct, yielded mixed results (33, 34) but it remains to be determined to what extend this relates to the biology, the therapeutic molecule, the trial design, and/or the target population. Collectively, it is therefore plausible that IL-23−independent pathways modulate the disease outcomes observed in AxSpA patients.

## Lessons Learned

In conclusion, the genetic, experimental, functional, and clinical studies on the role of IL-23 in ankylosing spondylitis have yielded a number of important lessons with broader relevance. First, the IL-23/IL-17 axis is not a linear “cascade.” Rather IL-23 and IL-17 display partially overlapping but also partially distinct biology and pathobiology. Second, with the exception of monogenic diseases with high penetrance and rare extreme phenotypes, human genetic and expression studies are great tools to create hypotheses but are not fit for purpose to proof or disprove these. Third, animal models are still essential to help us to understand the biology of a pathway but one should remember that they are pathway models and, unfortunately, only sporadically disease models. Fourth, the function of a pathway and even a single inflammatory mediator is highly context dependent. Therefore, the development and validation of disease-relevant functional models is today one of the most critical factors needed to secure a rapid and adequate translational of emerging insights in basic immunology into novel therapeutics.

## Data Availability Statement

The original contributions presented in the study are included in the article/supplementary material. Further inquiries can be directed to the corresponding author.

## Author Contributions

DB and IEA drafted this manuscript, reviewed the content, and approved the submitted version. All authors contributed to the article and approved the submitted version.

## Conflict of Interest

DB is currently an employee of UCB Pharma. IA received research grants from Pfizer, Tanabe Research Labs, Merck, and Novartis.
